# Prevalence of Zoonotic Gastrointestinal Helminth Parasite among Dogs in Suryabinayak, Nepal

**DOI:** 10.1155/2023/3624593

**Published:** 2023-05-30

**Authors:** Punya Ram Sukupayo, Semsal Tamang

**Affiliations:** ^1^Department of Zoology, Bhaktapur Multiple Campus, Tribhuvan University, Bhaktapur, Nepal; ^2^Central Department of Zoology, Tribhuvan University, Kathmandu, Nepal

## Abstract

Dogs are popular pets around the world and have always had a very close relationship with humans. Zoonotic gastrointestinal helminth parasites are a great threat to both stray and pet dogs. This study was carried out to determine the prevalence of zoonotic gastrointestinal helminths in dogs. 400 samples were collected, including 200 from pet dogs and 200 from stray dogs. The samples from pet dogs were collected from the ground immediately after voiding with the help of the owner, whereas stray dogs were caught by using a dog catcher, and the samples were collected directly from the rectum by using a gloved index finger. All collected samples were examined under a microscope using sedimentation and flotation techniques. The overall prevalence of infection was found to be 59.50%, with a significantly higher prevalence in stray dogs (70%) than that in pet dogs (49%). *Ancylostoma* spp., *Toxocara* spp., *Trichuris* spp., *Capillaria* spp., *Dipylidium caninum*, and *Taenia/Echinococcus* spp. were six different species found in the current study. The study showed the highest prevalence of *Ancylostoma* spp. (49.16%) and the least prevalence of *Capillaria* spp. (0.84%). In the age-wise study, puppies had a significantly high infection rate (86.96%). Similarly, we recorded a significantly higher prevalence of intestinal helminths among nondewormed pet dogs (78.65%) than among dewormed pet dogs (25.23%). This study highlights the severe environmental contamination shed by dogs, causing a higher risk of zoonotic transmission. It indicates the urgent need to manage these parasites in dogs and educate the public on how to care for their pets and the parasites they shed.

## 1. Introduction

Dogs are very popular pets around the world and have always had a very close relationship with humans. The gastrointestinal helminth parasites (GIHPs) are a great threat to both stray and pet dogs. Most of them are zoonotic parasites. More than 60 distinct zoonotic disease subtypes are associated with dogs. The majority of them pose a great threat to human health [1–5]. *Giardia, Cystoisospora, Taenia, Echinococcus, Dipylidium, Toxocara, Ancylostoma, Capillaria*, and *Trichuris* are some of the most common intestinal parasites that infect dogs [5, 6]. The most significant epidemiologically include *Toxocara, Ancylostoma*, and *Echinococcus* [3, 7]. *Echinococcus*, *Giardia*, *Toxocara*, and *Cryptosporidium* are common zoonotic GIHPs of dogs. They may spread to people causing different illnesses, including hydatidosis, giardiasis, toxocariasis, and cryptosporidiosis [8]. Stray and semidomesticated dogs have a greater frequency of parasites because they reside in lower resource habitat, which have more favourable environmental conditions for the growth of parasites [9]. Overcrowding and environmental contamination may also contribute to the spread and maintenance of parasitic infections among dogs. In order to manage parasitic infections in stray and semidomesticated dogs, specific treatment may be required [10]. The prevalence of gastrointestinal parasites among dogs is high in developing countries such as Nepal [11] because dogs in these countries are not or rarely treated for parasitic diseases and there is a lack of policies for pet ownership [12]. Therefore, dogs pose a serious threat to public health even though they are helpful pet animals [13], specially in developing countries.

The trend of raising dogs as pets is growing, and the number of stray dogs has also sharply grown in Suryabinayak municipality. All the stray dogs and most of the semidomesticated dogs wander and defecate freely on the streets and public areas. Moreover, the pet owners also bring their dogs in the public areas for pooping. Large numbers of infective stages (oocysts, cysts, eggs, and larvae) of intestinal parasites are excreted by infected dogs through their faeces and are left behind in parks, playgrounds, gardens, roadways, and other public areas, which pose a danger of infection to humans. Therefore, dogs serve as both reservoirs and transmitters of many parasites [14]. Transmission of these infective stages occur accidentally through the contaminated soil or by ingesting contaminated food, raw vegetables, or water or by direct contact with an infected dogs [3, 7]. Children are especially susceptible to the infections due to their frequent interactions with dogs, as well as the fact that they play frequently in open areas such as parks, playgrounds, public gardens, temples, and roadways with poor cleanliness standards [3, 15]. To reduce the hazards to humans, it is crucial to understand the epidemiology of zoonotic parasite diseases in close-knit animals such as dogs. There are very few reports available about the risk of dog-man infection in Nepal's rural and suburban regions. Many stray, semidomesticated, and domestic dogs wander freely in the majority of these localities with little to no veterinary care. The aim of this study is to assess the prevalence of zoonotic gastrointestinal parasites in dogs in Suryabinayak municipality, Nepal.

## 2. Materials and Methods

### 2.1. Study Area and Study Population

**Figure 1:**
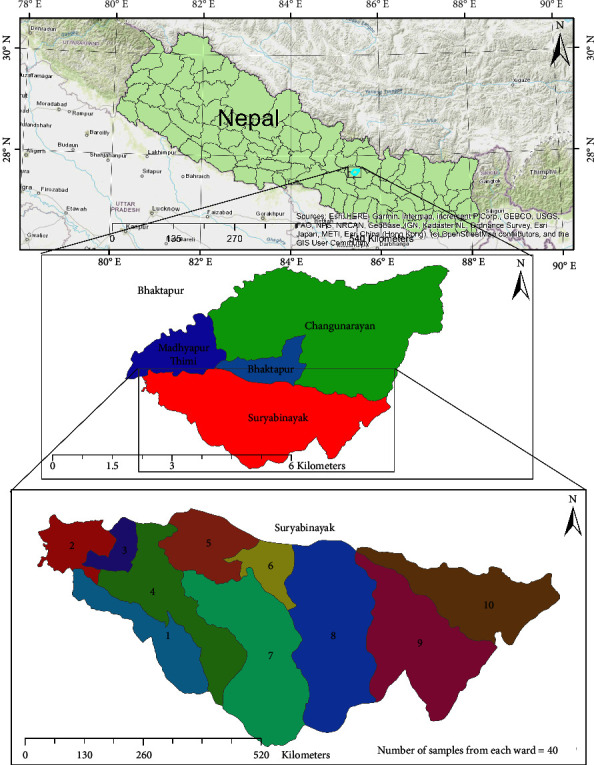
Study area.

The Suryabinayak municipality of central Nepal was the study area (Figure 1), which is located in the southern part of Bhaktapur district. The municipality has been divided into ten wards and covers 42.45 km^2^ area [16]. According to Census conducted by Central Bureau of Statistics (CBS), Suryabinayak municipality had total population of 140,085 [17]. Unfortunately, the official record of the dog population in the municipality was not available. However, it is common to see dogs in the streets in and around human settlement area and many households keep dogs as pets. Samples were collected from both stray dogs and pet dogs. For sample collection, 20 pet dogs and 20 stray dogs were selected from each ward. So, there were a total of 400 samples including 200 from pet dogs and same numbers from stray dogs. We gave red mark to the stray dog after collection of sample to avoid repetition. Stratified random sampling technique was used for the collection of samples. Pet dogs were classified into four age groups as puppies (0–6 months), young dogs (>6 months to 12 months), adults (>12 months to <10 years), and old (10 years and older) as per information obtained through interview with the owner.

### 2.2. Sample Size Determination

The formula was used to determine sample size [18] with a 95% confidence level. The expected prevalence of GIHPs in the dogs of Kathmandu was 46.7% [19].

(1) $$\begin{array}{c} n=\frac{1.96^{2}\;x\;p\;\left(1-p\right)}{d^{2}}\text{,} \end{array}$$

where *n* represents the required sample size; *p* represents the expected prevalence, i.e., 46.7%; and *d* represents the desired absolute precision, 5%.

The minimum sample size given by using the above formula was 383, but the final sample size was 400.

### 2.3. Questionnaire Survey

Different structured questionnaires were prepared and interviewed among pet owners. The questionnaires were set to assess the sex and age of pet dogs, deworming schedule, and awareness about canine intestinal parasitic zoonosis.

### 2.4. Sample Collection

The stray dogs were caught by using dog catcher and stool sample was taken directly from the rectum by using gloved index finger. Samples from pet dogs were collected from the ground instantly after pooping with the help of the owner. The purpose of the research work was shared with the pet owners, who were also instructed to collect stool samples from the ground instantly after pooping by using a polythene bag while they took out their dogs. All the collected samples were kept in appropriately labelled leak-proof containers and transported to the laboratory for immediate examination, and a 1-2% formalin solution was used for preservation whenever immediate examination was not possible. Total 400 samples were collected from the study area during the study period with an equal number of samples (*N* = 200) from both pet and stray dogs.

### 2.5. Faecal Examinations

The samples were first checked with naked eye for any adult stage of the parasites and then these were prepared for microscopic examination. Each faecal sample was examined qualitatively using the formal-ether sedimentation technique [20] and salt floatation techniques [21]. The result was considered positive if either any adult stage of the parasite was detected during the macroscopic examination, at least one parasite egg was found during the microscopic examination, or both [22]. The parasite stages and eggs of parasite were identified by using standard morphological criteria [23].

### 2.6. Statistical Data Analysis

Statistical analysis was performed by using R version 3.2.2. The Pearson's chi-square test was used to evaluate bivariate relationships between the result and specific explanatory factors. *P* < 0.05 was taken as statistically significant for all analysis.

## 3. Results

**Figure 2:**
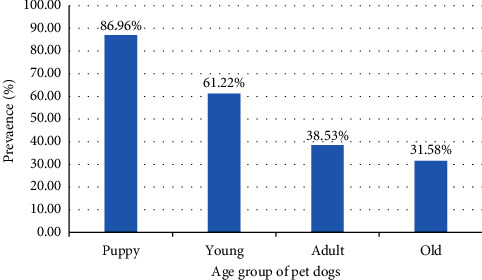
Age-wise prevalence of intestinal helminth parasites in pet dogs (*P* value = 3.54*e* − 5; *χ*^2^ = 23.28).

Present study revealed overall prevalence of infection with zoonotic GIHPs being 59.50% (238/400) (Table 1). Equal number of faecal samples were collected from both stray and pet dogs i.e., 200 from each. The significantly higher prevalence was found in stray dogs (70%) than that in pet dogs (49%). Among 400 samples, 243 were from male and 157 were from female dogs. The sex-wise prevalence showed higher infection of zoonotic GIHPs in male dogs (63.79% v/s 52.87%) but the sex was found statistically insignificant (*P* > 0.05). The males of both stray and pet dogs were found highly infected by zoonotic GIHPs. In the age-wise prevalence, the highest (86.96%) prevalence of intestinal helminths was found in puppies and the least (31.58%) was among the old dogs (Figure 2). The age of the dog was found to be statistically significant (*P* < 0.05).

**Figure 3:**
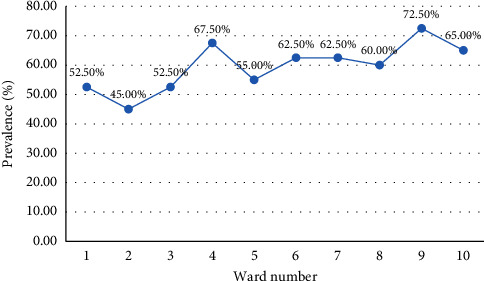
Ward-wise prevalence of intestinal helminth parasites (*P* value = 0.34; *χ*^2^ = 10.13).

Among the 200 samples collected from pet dogs, 111 were from dewormed dogs within six months, and the remaining 89 samples were from nondewormed pet dogs. The prevalence of gastrointestinal helminths was found significantly higher among samples from nondewormed pet dogs (78.65%) than from dewormed pet dogs (25.23%) (*P* < 0.05). The study revealed six different types of zoonotic GIHPs (*Ancylostoma* spp., *Toxocara* spp., *Trichuris* spp., *Capillaria* spp., *Dipylidium caninum*, and *Taenia/Echinococcus* spp.) belonging to two different classes (nematoda and cestoda). Among them, *Ancylostoma* spp. was the most common helminth (49.16%) parasite and *Capillaria* spp. was found to be the least prevalent (0.84%). The parasites of the class nematoda were found to be higher (73.95%) than cestoda (Table 2). The highest prevalence (72.50%) was found in ward number nine and the lowest (45.00%) prevalence was found in ward number two (Figure 3), but the ward was found statistically insignificant (*P* > 0.05).

### 3.1. Limitations of the Current Study

The type of anthelminthic drugs used and the type of food materials served to pet dogs were not investigated during this research work. Similarly, the teeth of the stray dogs were not studied, hence the age-wise prevalence of zoonotic GIHPs for the stray dogs was not calculated.

## 4. Discussion

The zoonotic GIHPs are a serious health problem among dogs in developing countries including Nepal and cause great threat to human health. Understanding the epidemiology of zoonotic parasitic infections in dogs helps to minimize the risk to humans. The overall prevalence of intestinal helminths found in the present study was found to be 59.5%. This result was comparable with earlier studies conducted in Rupandehi 58.75% [24]; Mampong, Ghana 52.60% [15]; Mazandaran, Iran 59.50% [1]; Nigeria 52.60% [25]; Guimaraes, Portugal 57.20% [26]; 56% in Sidama, Ethiopia [27]; and Italy 52.50% [28]. But the prevalence of present study was higher than the previous studies conducted in Kathmandu 46.7% [19], Lower Dir district, Pakistan 26.8% [29]; Tabasco, Mexico 19.20% [30]; Lodz, Poland 37.40% [3]; Hamadan, Iran 20.40% [31]; Thailand 40.10% [32]; La Habana, Cuba 43.90% [33]; Zaria, Nigeria 33.90% [34]; Iran 19.10% [35]; Osaka, Japan 39.20% [4]; Venezuela 35.50% [36]; and Czech Republic 17.60% [6]. Interestingly, the higher prevalence of GIHPs was recorded in India 88.9% [37]; more than 80% in Mexico [13, 38]; 80%–95.2% in Iran [39–41]; 76.27% in Ethiopia [42]; 83.60% in Brazil [22]; 71.60% in Catalonia, Spain [10]; 90% in Kandy, Peradeniya [11]; 98.60% in Italy [2]; 80% in Yucatan, Mexico [13]; and 90.70% in Uttar Pradesh, India [37]. These variations are probably due to differences in climate and geographical location. The prevalence of zoonotic helminths in stray dogs was higher (70%) than in pet dogs (49%), which is similar with the previous result from different parts of the globe [3, 19, 24, 29, 31, 34]. Stray dogs roam the open areas, exposing them to risk factors of disease transmission. The lack of anthelmintic treatment is another reason for the higher positivity in stray dogs.

The number of intestinal helminth parasite species registered in the survey (i.e., six) was within the range of 5–10 species documented worldwide [4, 19, 26, 37, 41]. *Ancylostoma* spp. had the highest prevalence, followed by *Toxocara canis*, which has zoonotic importance. This finding is also supported by the previous studies in Nepal [19, 24], India [13, 37], Ethiopia [27], and Mexico [13, 37]. Application of the One Health concept has to be encouraged to improve the management of intestinal helminth parasites and to reduce the risk of exposure for both dogs and humans. The study needs to be replicated in other districts of Nepal to give an overall variation of helminth infection among dogs. Sex-wise prevalence showed higher infection in male dogs than in females. A study from Rupundehi also showed similar findings [24]. The result is also supported by results obtained in Iran, Italy, India, and Mexico [28, 38, 41].

Although studies have divided the age of the host into several groups, there is universal agreement that puppies have a greater frequency of intestinal helminth parasites than adults. Horizontal transmission by the consumption of larvae from vertebrate or invertebrate paratenic hosts or the eating of embryonated eggs from the environment, as well as vertical transmission, trans-placental, and/or trans-mammary transmission, and other means infect puppies [7]. The parasite-specific immunity is often developed with age, most likely as a result of one or more encounters [36]. According to the current study, dogs under a year old were more than twice as likely as older dogs to get helminth parasites. Similar results were previously reported from Kathmandu and Rupandehi in Nepal [19, 24], Osaka in Japan [4], and in Nigeria [25]. The significantly higher prevalence of gastrointestinal helminths in nondewormed pet dogs (78.65%) than in dewormed pet dogs (25.23%) illustrates the effectiveness of anthelmintic usage in dogs. The highest positivity for zoonotic GIHPs was associated with a lack of knowledge about canine zoonotic diseases among local people, semidomestication of pet dogs, failure to provide anthelmintic drugs to pet dogs, and an open sanitary system. So, it was found different for different wards of Suryabinayak municipality. The highest prevalence (72.50%) was found in ward number nine and the lowest (45.00%) prevalence was found in ward number two. The highest prevalence of zoonotic GIHPs among the dogs of ward number nine might be due to lack of awareness among local people, carelessness of dog owner, practice of semidomestication of dogs, and an open sanitary system throughout the area of ward number nine. Whereas the lowest prevalence of zoonotic GIHPs in the dogs of ward number two might be due to proper management of the sanitary system and full domestication of pet dogs.

## 5. Conclusion

The present study noticeably verified that the most important zoonotic gastrointestinal helminths are present in both stray and pet dogs in Suryabinayak municipality, Nepal. They are great challenges for public health. The result indicates the necessity for a reduction in the number of stray dogs in Suryabinayak municipality in order to minimize risks of zoonotic gastrointestinal helminth parasitic infection to humans. Local authorities should implement effective strategies for homeless dog population control, such as the creation of new shelters with adequate veterinary care, large-scale sterilization of animals, deworming programs for both stray and pet dogs, as well as greater enforcement of laws concerning pet ownership and educating owners.

## Figures and Tables

**Table 1:** Prevalence of intestinal helminth parasites.

	Male dogs	Female dogs	Pet dogs	Stray dogs	Dewormed pet dogs	Nondewormed pet dogs	General
Total	243	157	200	200	111	89	400
Positive no. (%)	155 (63.79)	83 (52.87)	98 (49.00)	140 [70.00]	28 (25.23)	70 (78.65)	238 (59.50)
*P* value	0.063		2.97*e* − 5		1.72*e* − 13		
*χ * ^2^	3.46		17.44		54.30		

**Table 2:** Intestinal helminth parasites identified in the dogs of Suryabinayak municipality.

Class	Parasite	Dogs		Overall positive number (prevalence (%))
		Stray	Pet	
		No. (%)	No. (%)	
Nematoda	*Ancylostoma* spp.	67 (47.86)	50 (51.03)	117 (49.16)
	*Toxocara* spp.	36 (25.71)	16 (16.33)	52 (21.85)
	*Trichuris* spp.	5 (3.57)	0 (0.00)	5 (2.10)
	*Capillaria* spp.	2 (1.43)	0 (0.00)	2 (0.84)

Cestoda	*Dipylidium caninum*	8 (5.71)	20 (20.41)	28 (11.77)
	*Taenia/Echinococcus* spp.	22 (15.71)	12 (12.24)	34 (14.29)

## Data Availability

The data used to support the findings of this study are available from the corresponding author upon reasonable request.
